# Oral hygiene habits and possible transmission of COVID-19 among cohabitants

**DOI:** 10.1186/s12903-020-01274-5

**Published:** 2020-10-19

**Authors:** María José González-Olmo, Bendición Delgado-Ramos, Ana Ruiz-Guillén, Martín Romero-Maroto, María Carrillo-Díaz

**Affiliations:** 1 Dentistry Department, Rey Juan Carlos University, Avda de Atenas s/n, 28922 Alcorcón, Madrid, Spain; 2 Dentistry Department, Granada University, Campus de la Cartuja s/n, 18071 Granada, Spain

**Keywords:** Coronavirus, Coronavirus infections, COVID-19, Transmission, Oral hygiene, Toohbrushing

## Abstract

**Background:**

To find out whether misuse of dental hygiene, in terms of certain dental habits, may facilitate the spread of COVID-19 among cohabiting individuals.

**Methods:**

302 COVID-19 infected (PCR +) subjects cohabiting with someone else at home were selected for an observational cross-sectional study. An anonymous online questionnaire was developed using Google forms to avoid person-to-person contact. The structured questionnaire consisted of questions covering several areas: sociodemographic data, cross transmission to another person living together, oral hygiene habits during confinement, care and disinfection control behaviours in the dental environment like sharing toothbrush, sharing toothbrush container, sharing toothpaste, placing brush vertically, placing cap with hole for brush, disinfecting brush with bleach, closing toilet lid before flushing.

**Results:**

Tongue brushing was more used in the group where there was no transmission of the disease to other members (p < 0.05). Significant differences were found for shared toothbrush use (p < 0.05), although shared use was a minority in this group (4. 7%), significant differences were also found for the use of the same container (p < 0.01), shared use of toothpaste (p < 0.01), toothbrush disinfection with bleach (p < 0.01), brush change after PCR + (p < 0.05). The women performed significantly more disinfection with toothbrush bleach (p < 0.01), closing the toilet lid (p < 0.05) and changing the brush after PCR + (p < 0.05).

**Conclusions:**

The use of inappropriate measures in the dental environment could contribute to the indirect transmission of COVID-19 between cohabitants.

## Background

The new coronavirus (SARS-CoV-2) is causing concern in the medical community, as the virus is spreading globally. The fact that asymptomatic people are potential sources of infection [1] justifies a thorough analysis of the dynamics of the transmission of the current outbreak. The virus is mainly transmitted through direct or indirect contact with the mucous membranes of the eyes, nose or mouth [2, 3]. In this context, the detection of SARS-CoV-2 and a high viral load in the sputum of a convalescent patient raises concerns about the potential transmissibility after recovery.

The SARS-CoV-2 virus, commonly referred to as a coronavirus because of its unique appearance, has a glycoprotein configuration on its exterior, forming spicules, through which it binds to human cells. In order to protect its genetic contribution, it has a double layer made of lipids in its lower part that performs this protective function [4]. The SARS-CoV-2 virus infects human cells using the ACE2 receptors, which are widely distributed in the upper respiratory tract (hence the lung lesions it causes in affected people) and the epithelial cells lining the ducts of the salivary glands, these being early targets of infection [5–7]. They can also be in the mouth, mainly on the tongue, which is a great reservoir of viral germs. Therefore, tooth brushing, interproximal hygiene and tongue cleaning are essential in order to reduce the viral load in the oral area [3, 8].

In addition, in order to prevent cross-contamination, it is important to ensure that tooth brushes within the family are not in the same container. After use, cleaning devices become contaminated and, if not disinfected, can be a reservoir of microorganisms [9] (including bacteria, viruses and fungi) that maintain their viability for a significant amount of time, ranging from 24 h to 7 days. Microbial survival promotes the reintroduction of potential pathogens into the oral cavity or the spread to other individuals when cleaning devices are stored together or shared [10].

This has always been a bad idea, but today this separation has become a real necessity, as if we are asymptomatic carriers of the virus without knowledge of it and the brushes are placed together, it can encourage cross-contamination. Recent studies have observed that COVID-19, through friction with the oral mucosa, can be transmitted to the individual [11].

The same tube of toothpaste should also not be used between members of the same family, as this is another way of facilitating cross-contamination. It is also necessary to store the toothbrush with the brush head upwards, as this facilitates faster drying and hinders the spread of microorganisms [12–14]. Even if the brush is accompanied by a wrapper, it must have openings to facilitate drying.

Toilets should be considered as a possible source of viral contamination of indoor and surface air. In fact, constant microbial contamination of the indoor environment often occurs after toilet flushing, and this can be a major source of spread, not only for enteric but also for respiratory viruses, which are also often eliminated by faecal means. Toilet flushing generates a large number of droplets of different sizes: the larger droplets settle quickly on surrounding surfaces, while the smaller ones can be inhaled or remain in the air for a long time [15]. The level of contamination in the toilet environment has been studied, concluding that the highest levels of surface contamination were located near the source of the aerosol, at the level of the toilet seat. However, contaminated surfaces were also found at a distance of 83 cm from the toilet. This is the reason why the toothbrush should also be kept away from the toilet (at least 1 m) to avoid possible contamination, as the virus is also found in feces and urine [16, 17].

At the end of an eventual infectious, process it is necessary to be cautious and use a new brush, as even if the power of reinfestation of the virus is not known, it is necessary to bear in mind that the brush can constitute an emitter of germs to other brushes used by other members of the family or even to one’s self.

Disinfection of the brush head after use with povidone-iodine at 0.2% or hydrogen peroxide diluted at 1% for 1 min [18] is very necessary to maintain good cleanliness [19], as the brush filaments can be infected by germs from the environment. It is necessary to know and take into account the temporary duration of the stay of the coronavirus on different surfaces [18]; in order to be prevent infection, it is important to know that the duration for the stay of coronavirus on plastic is 72 h.

When there is an active development of COVID-19, a 0.2% povidone-iodine mouthwash or 1% dilution of hydrogen peroxide can be used for 1 min [18] to try to control the oral load of germs, as although scientific evidence is limited [1, 18], it has been observed that such products can be effective in rendering the lipid envelope of the virus inoperative.

There are many families who are currently confined to their homes because they are positive for COVID-19. Precautionary measures regarding cleanliness and asepsis to be performed in the homes by family members are well-known in order to prevent infection among them [20]. However, less emphasis has been placed on oral care to reduce the viral load and on the dental environment to prevent the risk of cross-contamination of COVID-19.

Taking into account the above considerations, the aim of this research is to find out whether misuse of dental hygiene, in terms of certain dental habits, may facilitate the spread of COVID-19 among cohabiting individuals.

## Methods

### Design type

This was a cross-sectional, observational study conducted in Spain for fifteen days (April 15–30 2020), four weeks after the start of the confinement in Spain.

### Data collection

These data collection efforts were particularly designed to avoid person-to-person contact. It was an online study, and only participants with Internet access could participate in the study. A snowball sampling technique was used. An anonymous online questionnaire was developed using Google forms with a consent form attached. The link to the questionnaire was sent by email, WhatsApp and other social networks through the researchers. Participants were encouraged to complete the survey with as many people as possible. Therefore, the link was forwarded to people apart from the first point of contact, etc.

Included participants were over 18 years old, able to understand Spanish, and willing to give an informed consent. A total of 2305 subjects agreed to the survey, but only those subjects who had a confirmation in PCR (Polymerase Chain Reaction) of a COVID-19 infection and who were living with another person with whom they shared a bathroom were selected, the sample being reduced to 302 subjects included in the analysis. The survey and consent to participate were approved by the King Juan Carlos University Ethics and Research Committee (Registration number: 0103202006520).

### Instruments

The structured questionnaire (included as supplementary file) consisted of questions covering several areas: (1) sociodemographic data (age, gender and educational level), (2) cross transmission to another person living in the same home and sharing a bathroom, with a response format carried out via a dichotomous question (yes = 1/no = 0), (3) oral hygiene habits during confinement (brushing 2 or more times per day, flossing once per day, mouth rinsing once per day, brushing tongue once per day). Responses were rated on a 5-point Likert scale ranging from 1 to 5, with “Never” = 1, “Almost never” = 2, “Sometimes” = 3, “Almost always” = 4 and “Always” = 5. Questions also covered (4) care and disinfection control behaviors in the dental environment (Usually sharing a toothbrush, usually sharing a toothbrush container, usually sharing toothpaste, usually placing brush vertically, usually placing cap with hole for brush, usually disinfecting brush with bleach, usually closing toilet lid before flushing, changing toothbrush after COVID-19 + test). The response format was carried out via a dichotomous question (yes/no).

### Statistical analysis

The study presents a cross-sectional descriptive study, in which the variables considered are those described in the previous section. Statistical analysis was performed using the SPSS version 24 (SPSS Inc., Chicago, IL, USA). Data analysis included descriptive statistics and the Kolmogorov–Smirnov test to evaluate the assumption of normality, which was confirmed. In order to know the possible differences between groups with infection from a single family member and those with an extension to more than one household member, *T* tests were performed in the case of quantitative variables and Chi-square tests in the case of variables. Significance levels were established at 0.05.

## Results

The sample consisted of 145 (48%) men and 157 (52%) women with an average age of 39.25 (± 9.94).

In terms of educational levels for the total sample, 34.1% had completed primary school, 29.8% had completed secondary school and 36.1% had obtained a university degree. 59.6% of the sample corresponds to a medium socio-economic level.

56.3% of the sample had a person living with them affected by COVID-19 and positive in a PCR test.

### Oral hygiene habits

Only 33.8% brushed their teeth 2 or more times every day, 20.2% flossed every day, 15.2% used a daily rinse and 17.2% brushed their tongue every day.

We found significant differences in oral hygiene measures for tongue brushing (t = 2.202; p = 0.029*). This hygiene measure was more used in the group in which there was no transmission of the disease to other members of the home. No significant differences in these measures were found in terms of sex. Table 1

**Table 1:** Mean, standard deviation and significance for the variables dental hygiene and disinfection control of dental environment by sex and cross infection group

Variables	Man	Woman	p	Cross infection	Non cross infection	p
	N =145	N = 157		N = 170	N = 132	
	M (SD)	M (SD)		M (SD)	M (SD)	
	N (%)	N (%)		N (%)	N (%)	
*Dental hygiene*						
Toothbrushing	3.4 (1.3)	3.6 (1.2)	0.304	3.5 (1.3)	3.6 (1.2)	0.541
Flossing	3.0 (1.3)	3.1 (1.3)	0.461	3.0 (1.3)	3.2 (1.3)	0.072
Oral rinse	2.8 (1.3)	3.0 (1.2)	0.107	2.8 (1.3)	3.0 (1.3)	0.323
Tongue-brushing	3.0 (1.2)	3.0 (1.3)	0.847	2.8 (1.2)	3.2 (1.3)	0.029*
*Environmental care and disinfection control*						
Shared toothbrush use						
Yes	4 (2.8%)	5 (3.2%)	0.828	162 (95.3%)	131 (99.2%)	0.045*
Not	141 (97.2%)	152 (96.8%)		8 (4.7%)	1 (0.8%)	
Sharing brush container						
Yes	99 (68.3%)	95 (60.5%)	0.159	127 (74.7%)	65 (50.8%)	0.000**
Not	46 (31.7%)	62 (39.5%)		43 (25.3%)	67 (49.2%)	
Sharing of toothpaste						
Yes	76 (52.4%)	76 (48.4%)	0.487	99 (58.2%)	53 (40.2%)	0.002**
Not	69 (47.6%)	81 (51.6%)		71 (41.8%)	79 (59.8%)	
Brush in vertical position						
Yes	120 (82.8%)	123 (78.3%)	0.334	143 (84.1%)	100 (75.8%)	0.069
Not	25 (17.2%)	34 (21.7%)		27 (15.9%)	32 (24.2%)	
Capped brush						
Yes	86 (59.3%)	75 (52.2%)	0.216	93 (54.7%)	75 (56.8%)	0.714
Not	59 (40.7%)	82 (47.8%)		77 (45.3%)	57 (43.2%)	
Brush disinfection with bleach						
Yes	6 (4.1%)	20 (12.7%)	0.008**	8 (4.7%)	18 (13.6%)	0.006**
Not	139 (95.9%)	137 (87.3%)		162 (95.3%)	114 (86.4%)	
Toilet lid closure						
Yes	43 (29.7%)	67 (42.7%)	0.019*	42 (24.7%)	68 (51.5%)	0.000**
Not	102 (70.3%)	90 (57.3%)		128 (75.3%)	64 (48.5%)	
Brush change after PCR + test						
Yes	115 (79.3%)	138 (87.9%)	0.043*	136 (80%)	117 (88.6%)	0.043*
Not	30 (20.7%)	19 (12.1%)		34 (20%)	15 (11.4%)	

### Care and control of disinfection of the dental environment

97% of the sample did not share the use of the toothbrush, but 64.2% used the same container to hold the toothbrushes, 50.3% used the same toothpaste, 80.5% put the toothbrush upright, 55.6% used a cap for the brush, only 8.6% of the sample dipped the brush in bleach after use, 36. 4% closed the toilet lid before flushing and only 16.2% did not change the brush after testing positive for PCR.

Significant differences were found between the group in which there was no intrafamily cross-transmission and in which there was cross-transmission for shared toothbrush use (x 2(1) = 4.006; p = 0.045*). Although shared use was a minority in this group (4.7%), significant differences were also found for the use of the same container (x 21) = 18.550; p = 0.000**), shared use of toothpaste (x 2(1) = 9.720; p = 0.002**), toothbrush disinfection with bleach (x 2(1) = 7.532; p = 0.006**), toilet lid closure (x 2(1) = 23.062; p = 0.000**) and brush change after PCR + (x 2(1) = 4.077; p = 0.043*). See Table 1.

When the association between the variables of oral hygiene and care and control of disinfection of the dental environment was explored, significant differences were found between the subjects who performed brushing hygiene with bleach and those who did not with respect to the use of dental floss (p = 0.028*) and tongue hygiene (p = 0.035*). Differences were also found between subjects who lowered the toilet seat and subjects who had tongue hygiene (p = 0.020*). Table 2

**Table 2:** Mean, standard deviation and significance for the variables disinfection control of dental environment and dental hygiene

	*Dental hygiene* variables							
	Toothbrushing		Flossing		Oral rinse		Tongue-brushing	
	M (SD)	p	M (SD)	p	M (SD)	p	M (SD)	p
*Environmental care and disinfection control*								
Shared toothbrush use								
Yes	3.6 (1.5)	0.848	3.4 (1.4)	0.518	2.6 (1.1)	0.458	3.1 (1.4)	0.863
Not	3.5 (1.2)		3.1 (1.3)		2.9 (1.3)		3.0 (1.2)	
Sharing brush container								
Yes	3.5 (1.3)	0.277	3.0 (1.3)	0.477	2.9 (1.3)	0.641	3.0 (1.2)	0.638
Not	3.6 (1.2)		3.2 (1.3)		3.0 (1.2)		3.0 (1.3)	
Sharing of toothpaste								
Yes	3.5 (1.3)	0.502	3.0 (1.3)	0.519	2.9 (1.3)	0.933	2.9 (1.2)	0.370
Not	3.6 (1.2)		3.1 (1.3)		2.9 (1.2)		3.0 (1.2)	
Brush in vertical position								
Yes	3.5 (1.3)	0.393	3.0 (1.3)	0.160	2.9 (1.3)	0.103	3.0 (1.2)	0.722
Not	3.6 (1.2)		3.3 (1.3)		3.1 (1.1)		3.0 (1.4)	
Capped brush								
Yes	3.6 (1.3)	0.143	3.2 (1.3)	0.119	2.9 (1.3)	0.644	3.1 (1.2)	0.08
Not	3.4 (1.2)		2.9 (1.3)		2.9 (1.3)		2.8 (1.3)	
Brush disinfection with bleach								
Yes	3.8 (1.0)	0.264	3.6 (1.0)	0.028*	2.9 (1.2)	0.973	3.5 (1.3)	0.035*
Not	3.5 (1.3)		3.0 (1.3)		2.9 (1.3)		2.9 (1.2)	
Toilet lid closure								
Yes	3.6 (1.2)	0.286	3.0 (1.2)	0.335	3,0 (1.2)	0.514	3.2 (1.2)	0.020*
Not	3.5 (1.3)		2.9 (1.3)		2,9 (1.3)		2.8 (1.2)	
Brush change after PCR + test								
Yes	3.6 (1.2)	0.234	3.1 (1.3)	0.703	2.9 (1.2)	0.671	3.0 (1.2)	0.445
No	3.3 (1.4)		3.0 (1.5)		2.8 (1.3)		2.8 (1.2)	

### Gender differences for the study variables

The differences in these measures in terms of gender were significant for personal hygiene or disinfection measures, such as disinfection with toothbrush bleach (x 2(1) = 7.087; p = 0.008**), closing the toilet lid (x 2(1) = 5.518; p = 0.019*) and changing the brush after PCR + (x 2(1) = 4.090; p = 0.043*). These measures were used more in women than in men with a significant difference.

## Discussion

In this study, we explored the role played by the correct use of anti-contamination measures in the dental environment to prevent infection among people living in the same house. The results have highlighted this association, considering that sharing a toothbrush, toothpaste, the same container for the brush, closing the toilet lid before flushing and changing the brush after the viral process could be a possible route of cross-contamination of COVID-19. However, when studying oral hygiene habits, no significant differences were found between the groups except for tongue cleaning. This result can be interpreted to indicate the tongue as the main oral organ acting as a reservoir of COVID-19 [5] and the importance of brushing to decrease the viral load of the individual carrier.

The study shows significant differences in the measures taken to avoid cross-contamination with respect to gender, with the figures being higher in women than men. This finding is consistent with previous results obtained in the literature regarding care and cleaning in the home, in which the leading role of women is emphasized. In addition, men seem to be more affected by COVID-19 than women [7, 20, 21], so it is doubtful whether this could be due to less comprehensive compliance with prevention measures.

It is also important to recognize some limitations of this study. First, a more definitive method would have been to measure the aerosol and surface viability of SARS-CoV-2 on the different surfaces and toilet environment but it is not possible because of the impossibility to visit each home due to the lockdown situation. Second, it is a matter of convenience. However, the sample size is acceptable to show a first approximation of what could happen if adequate measures are not taken in the dental environment. A possible third limitation comes from the use of self-report measures, which may be affected by responses based on social desirability. Finally, only measures affecting the dental environment have been considered, so the results could be partially biased.

This research has some relevant implications for the possible spread of COVID-19. There is evidence that daily hygiene measures are a vital part of infection prevention, which is important in the prevention of transmission and acquisition. There is evidence that everyday hygiene measures are a vital part of infection prevention and are important in preventing the transmission and acquisition of infection. Adopting a specific hygiene approach in our homes and our daily life (e.g., workplaces, public transport, gyms, nursery schools and shopping centers), in situations in which there is usually no mandatory hygiene policy, offers a way to maximize protection against infections.

In order to minimize the risk of viral infection among cohabitants, the population should be informed of the measures in the dental environment that should be taken to reduce possible cross-contamination, including not sharing a toothbrush or the same toothpaste tube, not sharing the cup where the toothbrush is stored, closing the toilet lid before flushing, disinfecting the toothbrush after each use and changing the toothbrush after a viral process.

If effectively implemented, hygiene in the home and in daily life has the potential to reduce infection rates and antibiotic consumption, thus reducing the selective pressure for the development and further spread of resistance [14]. As noted in recent global efforts to contain the SARS-CoV-2 virus and slow the spread of COVID-19, hygiene practices, including hand washing, are the first line of defense to reduce the transmission of infection. It is also important to recognize that while hygiene measures and disinfection of toilets and oral equipment to prevent the spread of COVID-19, appear to be necessary to consider in preventing the spread of COVID-19, it is vitally important to comply with all general measures outlined at the global level in order to contain the spread.

Although there is evidence that hygiene in the dental environment is important to prevent transmission of COVID-19 colonization and infection, further research is needed to demonstrate the extent to which poor hygiene in the dental environment may contribute to the burden of infection and cross-contamination of COVID-19. In addition, it would be interesting to know the different effects depending on the number of people in the household.

## Conclusion

The use of inappropriate measures in the dental environment could contribute to the indirect transmission of COVID-19 between cohabitants.

## Supplementary information

Additional file 1: Questionnaire of oral hygiene habits used for research in Spanish and translated into English.

## Data Availability

The datasets generated during and/or analysed during the current study are available from the corresponding author on reasonable request.

## Abbreviation

PCR: Polymerase chain reaction
