# Supplementary material for: Oral hygiene habits and possible transmission of COVID-19 among cohabitants
Source: BMC Oral Health. 2020 Oct 19;20:286. doi: 10.1186/s12903-020-01274-5 (PMC7569355; doi:10.1186/s12903-020-01274-5)
Supplement: Supplementary file 1 — Questionnaire of oral hygiene habits used for research in Spanish and translated into English. [file 12903_2020_1274_MOESM1_ESM.docx]

**Cuestionario de higiene oral durante el confinamiento (Versión en español)**

1. ¿Tienes 18 años o más?

(Por favor, marque sólo una de las siguientes opciones)

Si

No

2. ¿Ha sufrido de COVID-19?

(Por favor, marque sólo una de las siguientes opciones)

Sí, confirmado por una prueba de PCR.

Sí, he sufrido los típicos síntomas de COVID-19: fiebre, tos y falta de aliento, pero sin la confirmación de una prueba de PCR.

No, confirmado por una prueba de PCR.

No, no he sufrido los típicos síntomas de COVID-19 - fiebre, tos y falta de aliento, pero sin una confirmación de la prueba PCR.

3. ¿Vive con otra persona con la que comparte el baño?

(Por favor, marque sólo una de las siguientes opciones)

Si

No

Sólo los participantes que respondieron "Sí" a la primera pregunta, "Sí, confirmado por una prueba de PCR" a la segunda pregunta y "Sí" a la tercera pregunta completaron las siguientes preguntas. Se agradeció a todos los demás su participación y se cerró el cuestionario.

4. Edad: ______

5. Género

(Por favor, marque sólo una de las siguientes opciones)

Mujer Hombre

6. Nivel educativo:

(Por favor, marque sólo una de las siguientes opciones)

Sin estudios

Primaria

Secundaria

Universidad

7. ¿Alguien con quien vives y compartes el baño ha sufrido COVID-19?

(Por favor, marque sólo una de las siguientes opciones)

Si

No

8. **Hábitos de higiene oral durante el encierro**:

¿Se cepilló los dientes dos o más veces al día?

(Por favor, marque sólo una de las siguientes opciones)

Nunca

Casi nunca

A veces

Casi siempre

Siempre

¿Usaste el hilo dental al menos una vez al día?

(Por favor, marque sólo una de las siguientes opciones)

Nunca

Casi nunca

A veces

Casi siempre

Siempre

¿Te enjuagaste la boca al menos una vez al día?

(Por favor, marque sólo una de las siguientes opciones)

Nunca

Casi nunca

A veces

Casi siempre

Siempre

¿Te cepillaste la lengua al menos una vez al día?

(Por favor, marque sólo una de las siguientes opciones)

Nunca

Casi nunca

A veces

Casi siempre

Siempre

**9. Cuidado y control de la desinfección en el ambiente dental durante el encierro.**

¿Sueles compartir tu cepillo de dientes con la persona que comparte el baño?

Si

No

¿Sueles compartir el mismo recipiente de cepillo de dientes con la persona que comparte el baño?

Si

No

¿Sueles compartir el mismo tubo de pasta de dientes con la persona que comparte el baño?

Si

No

¿Sueles colocar el cepillo de dientes en posición vertical?

Si

No

¿Sueles colocar un tapón con agujeros para el cepillo de dientes?

Si

No

¿Sueles desinfectar el cepillo de dientes con lejía?

Si

No

¿Sueles cerrar la tapa del inodoro antes de tirar de la cadena?

Si

No

¿Cambiaste el cepillo de dientes después de una prueba de PCR de Covid-19 positivo?

Si

No

**Oral Hygiene during lockdown questionnaire (English version)**

1. Are you 18 or older?

(Please check only one of the following options)

Yes

No

2. Have you suffered from COVID-19?

(Please check only one of the following options)

Yes, confirmed by a PCR test.

Yes, I have suffered the typical symptoms of COVID-19 – fever, coughing, and shortness of breath, but without a PCR test confirmation.

No, confirmed by a PCR test.

No, I have not suffered any typical symptoms of COVID-19 – fever, coughing, and shortness of breath, but without a PCR test confirmation.

3. Do you live with another person with whom you share a bathroom?

(Please check only one of the following options)

Yes

No

Only participants who answered "Yes" to the first question, "Yes, confirmed by a PCR test" to the second question and “Yes” to the third question completed the following questions. Everyone else was thanked for their participation and the questionnaire was closed.

4. Age

5. Gender

(Please check only one of the following options)

Female Male

6. Educational level:

(Please check only one of the following options)

None

Middle School

High School University

7. Has someone who you live with and share a bathroom suffered COVID-19?

(Please check only one of the following options)

Yes

No

8. Oral Hygiene habits during the lockdown:

Did you brush your teeth 2 or more times a day?

(Please check only one of the following options)

Never

Almost never Sometimes

Almost always

Always

Do you floss at least once a day?

(Please check only one of the following options)

Never

Almost never Sometimes

Almost always

Always

Did you rinse your mouth at least once a day?

(Please check only one of the following options)

Never

Almost never Sometimes

Almost always

Always

Brushed your tongue at least once a day?

(Please check only one of the following options)

Never

Almost never Sometimes

Almost always

Always

9. Care and disinfection control behaviors in the dental environment during the lockdown.

Do you usually share your toothbrush with the person who shares the bathroom?

Yes

No

Do you usually share the same toothbrush container with the person who shares the bathroom?

Yes No

Do you usually share the same toothpaste tube with the person who shares the bathroom?

Yes No

Do you usually place the toothbrush vertically?

Yes No

Do you usually place a cap with holes for the toothbrush?

Yes

No

Do you usually disinfect the toothbrush with bleach?

Yes

No

Do you usually close the toilet lid before flushing?

Yes

No

Did you change the toothbrush after a Covid-19 positive PCR test?

Yes

No
